# Syncytiotrophoblast Vesicles Show Altered micro-RNA and Haemoglobin Content after *Ex-vivo* Perfusion of Placentas with Haemoglobin to Mimic Preeclampsia

**DOI:** 10.1371/journal.pone.0090020

**Published:** 2014-02-27

**Authors:** Tina Cronqvist, Karen Saljé, Mary Familari, Seth Guller, Henning Schneider, Chris Gardiner, Ian L. Sargent, Christopher W. Redman, Matthias Mörgelin, Bo Åkerström, Magnus Gram, Stefan R. Hansson

**Affiliations:** 1 Division of Obstetrics and Gynecology, Department of Clinical Sciences, Lund University Hospital, Lund University, Lund, Sweden; 2 Department of Clinical Pharmacology, Ernst Moritz Arndt University of Greifswald, Greifswald, Germany; 3 Department of Zoology, University of Melbourne, Melbourne, Australia; 4 Department of Obstetrics, Gynecology, and Reproductive Sciences, Yale School of Medicine, New Haven, Connecticut, United States of America; 5 Department of Obstetrics and Gynecology, Inselspital, University of Bern, Bern, Switzerland; 6 Nuffield Department of Obstetrics and Gynaecology, University of Oxford, John Radcliffe Hospital, Oxford, United Kingdom; 7 Division of Infection Medicine, Department of Clinical Sciences, Lund University, Lund, Sweden; Medical Faculty, Otto-von-Guericke University Magdeburg, Medical Faculty, Germany

## Abstract

**Background:**

Cell-free foetal haemoglobin (HbF) has been shown to play a role in the pathology of preeclampsia (PE). In the present study, we aimed to further characterize the harmful effects of extracellular free haemoglobin (Hb) on the placenta. In particular, we investigated whether cell-free Hb affects the release of placental syncytiotrophoblast vesicles (STBMs) and their micro-RNA content.

**Methods:**

The dual *ex-vivo* perfusion system was used to perfuse isolated cotyledons from human placenta, with medium alone (control) or supplemented with cell-free Hb. Perfusion medium from the maternal side of the placenta was collected at the end of all perfusion phases. The STBMs were isolated using ultra-centrifugation, at 10,000×g and 150,000×g (referred to as 10K and 150K STBMs). The STBMs were characterized using the nanoparticle tracking analysis, identification of surface markers and transmission electron microscopy. RNA was extracted and nine different micro-RNAs, related to hypoxia, PE and Hb synthesis, were selected for analysis by quantitative PCR.

**Results:**

All micro-RNAs investigated were present in the STBMs. Mir-517a, mir-141 and mir-517b were down regulated after Hb perfusion in the 10K STBMs. Furthermore, Hb was shown to be carried by the STBMs.

**Conclusion:**

This study showed that Hb perfusion can alter the micro-RNA content of released STBMs. Of particular interest is the alteration of two placenta specific micro-RNAs; mir-517a and mir-517b. We have also seen that STBMs may function as carriers of Hb into the maternal circulation.

## Introduction

Preeclampsia (PE) is a disorder that affects 2–7% of all pregnancies [1] and is characterized by new onset hypertension and proteinuria [2]. There is no method to predict the disorder [1]. To date, the only cure is delivery and the treatment is purely symptomatic [3]. PE is thought to develop in two stages; the first is initiated by defective placentation resulting in inadequate formation of the utero-placental circulation. This results in an inadequately perfused placenta, which causes reperfusion injury, oxidative stress and formation of reactive oxygen species (ROS). As a result of this, in the second stage, placenta derived factors are released into the maternal circulation where they are believed to cause systemic inflammation, endothelial damage and organ failure [4].

Recently, cell-free foetal haemoglobin (HbF) was described to be an important placenta derived factor, potentially linking the first and second stage of PE. Analysis of placental gene expression by Centlow et al. revealed that the haemoglobin (Hb) chains, Hbα2, Hbγ and Hbβ, were significantly up regulated in PE, and an accumulation of HbF in the vascular lumen of PE placentas [5]. Also, perfusion of the placenta with cell-free Hb was shown to induce structural damage similar to that seen in PE [6]. As both HbF and its down-stream metabolites methaemoglobin, free heme and ROS are potent redox agents that can cause tissue damage [7], it may be hypothesized that cell-free HbF plays a role in the aetiology of PE by inducing oxidative damage to the blood-placenta barrier [6]. Placenta derived factors, including HbF, then leak into the maternal circulation where they are able to contribute to oxidative stress, endothelial damage, inflammation, hypertension and proteinuria [8], [9]. Clinical studies have shown that HbF leaks into the maternal circulation as early as the first trimester and is increased in women that will subsequently develop PE [10]. Furthermore, the levels of HbF correlates with the blood pressure, i.e. the severity of the disease, in term pregnancies [8].

Programmed cell death leads to cell blebbing, causing apoptotic debris, and extracellular vesicles (EVs) to be released [11]. Circulating EVs are often divided into apoptotic debris, microvesicles (>100 nm) and exosomes (<100 nm), which in addition to their size, differ regarding their membrane proteins and mode of release [12]–[15]. The EVs are recognized as a form of cell-to-cell communication that can transport proteins, DNA, RNA and micro-RNA (miRNA) from one cell to another and alter the phenotype and response of target cells [16]–[22].

The placental syncytiotrophoblast cells release EVs, named syncytiotrophoblast vesicles or STBMs (previously called syncytiotrophoblast microparticles). They have been suggested to be important for the foetal-maternal cross talk, allowing the maternal immune system to adapt to the on-going pregnancy [14], [23]. The role of STBMs in the aetiology of PE is an emerging field of interest. The number of STBMs in maternal plasma increases significantly in PE compared to normal pregnancies [9], [16], [24]. Placental perfusion with cell-free Hb increases blebbing of the cell membranes suggesting an increased vesicle release [6]. Release of apoptotic material into the maternal circulation has been suggested to contribute to the endothelial dysfunction seen in PE and increased numbers of STBMs to be involved in the characteristic maternal inflammatory response [11], [25]–[27]. For example, studies have shown that STBMs isolated from perfused placentas, when incubated with cultured monocytes, up regulate CD54 and down regulate CD11a expression [26], and STBMs incubated with cultured human umbilical vein endothelial cells activate peripheral blood leukocytes [28], including both monocytes [23] and neutrophils [29].

Micro-RNA (miRNA) are small non-coding RNA molecules predicted to regulate approximately 30% of all human genes [30]. Gene expression is generally down regulated by miRNA, either by degradation of the target mRNA or by preventing its translation [31]. The miRNAs are important for the development and function of the placenta. There is an abundance of miRNAs in the placenta, originating from a large, primate-specific, genomic cluster commonly referred to as the chromosome 19 miRNA cluster (C19MC) [32], [33]. C19MC miRNAs are differentially expressed in trophoblastic cells, as well as placental tissue when comparing first and third trimester placentas [34], [35]. Placental trophoblasts have been shown to release exosomes containing miRNAs in general and to be enriched in miRNAs belonging to C19MC in particular [32], [33], [36]. Both pregnancy specific [37] and placenta-specific miRNA have been detected in maternal plasma [36]. Many groups have also reported differentially expressed miRNAs in PE and hypoxic placentas [38]–[41].

Previous work has described placental *ex-vivo* perfusion with cell-free Hb as a model for PE [6]. The aim of this study was to further investigate the harmful mechanisms of extracellular Hb, and to examine the characteristics of STBMs released from placentas perfused with cell-free Hb. In particular, we investigated the miRNA content of released STBMs, following perfusion with cell-free Hb. Nine interesting miRNAs were chosen for the study. We selected mir-222, mir-16 and mir-210 based on previous studies showing their involvement in the regulation of HbF expression [42]–[44] as well as their involvement in PE [38]–[41]. Mir-517a, mir-517b and mir-518b were chosen because of their placenta specificity [36] and mir-518b being dys-regulated in PE [41]. Mir-424 and mir-205 are altered in hypoxia [45] and mir-141 in PE [39].

## Materials and Methods

### Ethics statement

The ethical review committee at Lund University approved the study and all mothers gave their written informed consent before delivery.

### Placental perfusion and sample collection

Sample collection and dual *ex-vivo* perfusion of isolated human placental cotyledons was performed as previously described by May et al [6]. Briefly, the perfusion experiment consisted of three perfusion phases lasting 120 minutes each, with medium exchange between the phases. Perfusion medium was supplemented with cell-free adult Hb (HbA) in the foetal circulation to mimic the PE condition, during phase II (3 mg/ml HbA, n = 6) and medium only in phase I and III. Control experiments were performed using medium alone for all phases (n = 6). The perfusate was collected from the maternal side at the end of all phases and used for isolation and analysis of STBMs.

### Isolation of STBMs from the perfusion medium

The STBM isolation was performed according to established protocols [46] from phase I and II. Thirty five ml of the maternal perfusate was centrifuged twice at 1500×g for 10 minutes in order to remove cellular debris. Ten ml of the supernatant was then further centrifuged for 30 minutes at 10,000×g at 4°C (pellet referred to as 10K STBM). The supernatant was ultra-centrifuged for 2 hours at 150,000×g at 4°C (pellet referred to as 150K STBM). The 10K and 150K STBM pellets were washed once with 1xPhosphate Buffered Saline (PBS) and re-suspended in 150 µl and 50 µl PBS respectively, aliquoted and stored at −80°C.

### Characterization of STBMs in the perfusion medium

#### Protein concentration of STBMs

The STBM protein concentrations were determined spectrophotometrically using a NanoDrop Spectrophotometer ND-1000 (NanoDrop technologies, Wilmington, USA).

#### Transmission electron microscopy (TEM) of STBMs

Transmission electron microscopy (TEM) was performed in two sets of preparations, first with antibodies against the human proteins tissue factor (TF), CD 63 and hsa-mir-222, a micro-RNA Assay primer (Applied Biosystems Inc., Foster City, CA, USA), labelled with colloidal gold (30, 15 and 5 nm in diameter, BBI International) as previously described [47]. In the second preparation, antibodies against the human adult Hb (HbA) protein was labelled with colloidal gold. The STBMs from both the 10K and 150K fraction were mixed with gold-labelled conjugates for 20 minutes at room temperature and then processed for negative staining, as previously described [48]. It is known that vesicles are permeabilized during TEM preparation, allowing antibodies and probes to label targets both on the surface and inside the STBMs. TEM was carried out three times in different specimens for both control and Hb perfused STBMs.

#### Nanoparticle Tracking Analysis

Nanoparticle tracking analysis (NTA) was performed using the NanoSight NS500 instrument (NanoSight, Amesbury, UK)[49]. This instrument passes a focused 488 nm laser beam through a suspension of the particles of interest and collects the scattered light using conventional microscope optics aligned at 90° to the beam axis. An electron multiplying charge coupled device captures a video of the field of view at 30 frames per second. The NTA program identifies and tracks Brownian motion of each particle from frame to frame, thus enabling the calculation of the hydrodynamic diameter via the Stokes-Einstein equation.

Samples analysed with NTA were from the different phase II STBM preparations; 10K control STBM preparations (n = 6), 10K Hb (n = 6), 150K control (n = 6) and 150K Hb (n = 6). The STBM preparations were diluted in sterile filtered PBS at 1∶500 or 1∶1000 prior to analysis, in order to give vesicle counts of 1.5–9.0×10^8^/ml. The diluted sample was introduced into the sample chamber and ten 20-second videos were recorded (shutter speed of 600; camera gain of 250). Fresh sample was introduced automatically between each video recording to eliminate settling and reduce sampling error. The videos were processed using optimised instrument settings (detection threshold 10; blur automatic; and minimum particle size 100 nm).

### Analysis of miRNA in STBMs

#### RNA isolation

Small RNA was isolated from the10K and 150K STBMs using mirVana™ miRNA Isolation Kit (Applied Biosystems, Carlsbad, USA) according to manufacturer's instruction. Briefly, the RNA extraction procedure consists of a step using Acid-Phenol:Chloroform, separating RNA in an upper organic phase from RNA and proteins which partitions in a lower aqueous phase. This prevents the Hb protein from being present in the RNA preparations and interacting in the subsequent PCR procedure. All RNA sample concentrations were spectrophotometrically determined using a NanoDrop Spectrophotometer ND-1000 (NanoDrop technologies, Wilmington, USA). RNA quality and miRNA content was assessed with an Agilent 2100 Bioanalyzer, using the Small RNA assay (Agilent Technologies, Palo Alto, USA).

#### cDNA synthesis and real-time quantitative PCR

RNA was transcribed using TaqMan® MicroRNA Reverse Transcription Kit according to manufacturer's instructions (Applied Biosystems Inc., Foster City, CA, USA). 10 ng RNA was used for the10K STBMs. For the 150K STBMs, 5 ng RNA was used because of a lower RNA yield. The following nine miRNAs were analysed using pre-designed TaqMan® MicroRNA assays (Applied Biosystems): homo sapiens-microRNA-517b (hsa-mir-517b), hsa-mir-518b, hsa-mir-222, hsa-mir-424, hsa-mir-210, hsa-mir-16, hsa-mir-141, hsa-mir-205, hsa-mir-517a, Rnu6b. Sequence for the hsa-mir-517b assay corresponds to ppy-mir-517b (pongo pygmaeus-microRNA, according to the miRNA database mirbase.org), which differs two nucleotides from the human hsa-mir-517b-3p.

Quantitative PCR (qPCR) was performed using standard protocol supplied by manufacturer for TaqMan® MicroRNA Assays on an ABI PRISM 7000 sequence detection system (Applied Biosystems). Primers and probes as described above. Each reaction was run in duplicate. Negative controls with no template as well as no reverse transcriptase controls were included for every miRNA primer pair. Data were normalized to Rnu6b, commonly used in miRNA PCR procedures. The fold-change values were calculated by normalizing against control samples from control perfused placentas placentas.

### Statistical analysis

All statistical analysis was performed using Origin 9 software (Microcal, Northampton, MA, USA). Mann-Whitney *U*-test was used and p-value <0.05 was considered statistically significant.

## Results

### Protein concentration and RNA content of the STBMs

After isolating the 10K and 150K STBMs, protein concentration was determined (Table 1). Protein concentration in the 10K STBM controls was significantly higher than in the 150K STBM controls (p = 0.0022). There was a slight difference, however not significant, between 10K and 150K STBMs from the Hb perfusions (p = 0.0649). No difference was found between control and Hb perfusion in the 10K (p = 0.1320) or 150K STBMs (p = 0.5887).

**Table 1:** RNA and protein concentrations, as well as RNA/STBM ratio.

	Control	Hb
Small RNA concentration (ng/µl)		
Ph I 10K STBM	6,35±6,42	1,63±1,14
Ph II 10K STBM	17,3±6,48	16,85±1,41
Ph I 150K STBM	*	*
Ph II 150K STBM	4,14±3,01	7,41±3,32
Protein concentration (µg/µl)		
Ph I 10K STBM	3,17±2,11	1,34±0,91
Ph II 10K STBM	2,96±1,55	1,77±1,65
Ph I 150K STBM	0,76±0,39	0,67±0,21
Ph II 150K STBM	0,55±0,33	0,62±0,33
RNA/STBM ratio		
Ph I 10K STBM	1,19±1,32	2,40±3,05
PhII 10K STBM	6,52±2,30	15,92±9,9
Ph I 150K STBM	-	-
Ph II 150K STBM	8,04±3,88	14,09±7,28

Values are expressed as mean ± standard deviation.

*  =  immeasurable concentration by NanoDrop, RNA/STBM ratio not possible to calculate.

Analysis of the small RNA, with Agilent 2100 Bioanalyzer, confirmed the presence of miRNA in all samples. The small RNA concentrations (Table 1) in the two STBM fractions were expressed either as RNA concentration (ng/µl) or amount of RNA per mg of protein (RNA/STBM ratio) (Table 1). Clearly, there was more RNA in the 10K STBM pellets compared to the 150K STBM pellets (p = 0.0022). No difference was seen between the control and Hb perfusions within the same centrifugation group. After calculating the RNA/STBM ratio, adjusting for size and amount of STBMs, the levels of RNA appeared to increase after Hb perfusion (Table 1). The RNA/STBM ratio was also more variable after Hb perfusions compared to controls. However, these results never reached statistical significance, p = 0.0649 for 10K and p = 0.1320 for 150K.

### Characterization of STBMs

#### Transmission electron microscopy of STBMs

**Figure 1:**
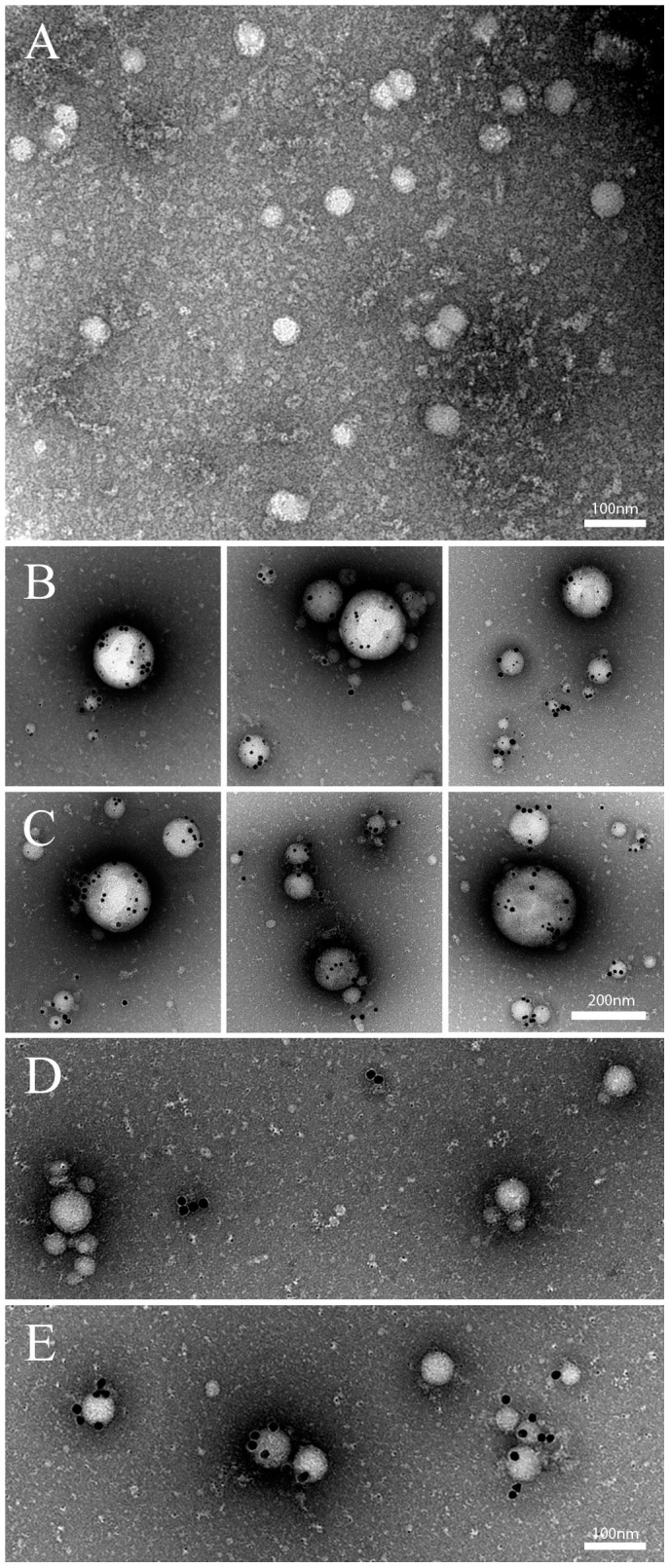
Transmission electron micrographs of isolated STBMs. The TEM panel (A) shows a selection of the 150K control STBMs, prior to gold-labelling. The STBMs were mixed with gold-labelled antibodies, as described in the Materials and Methods section, in two preparations. In the first preparation, STBMs shown in panels (B, control) and (C, Hb perfusions), were treated with antibodies against TF, CD63 and hsa-mir-222 labelled with colloidal gold of different sizes; CD63 (30 nm colloidal gold), TF (15 nm) and mir-222 (5 nm). Panel (B) shows the 150K control STBMs which are similar to the 150K Hb STBMs (C) in regards to the number of vesicles and their size. The 150K control STBMs (B) contain more mir-222 than 150K Hb STBMs (C). For the second preparation, STBMs shown in panels (D, control) and (E, Hb perfusions), were treated only with antibodies against Hb, labelled with colloidal gold. Control STBMs (D) carried small amounts of Hb, whereas STBMs from the Hb perfused placentas (E) showed higher labelling for Hb on particles of all sizes.

In order to distinguish between different sizes and types of vesicles, STBMs were investigated with transmission electron microscopy (TEM) using CD63 as a marker for exosomes [14] and TF as a general STBM marker [50] (Figure 1). Samples from both the 10K and 150K fractions as well as samples from both control and Hb perfusions, contained vesicles that were marked with CD63, TF and both (Table 2, and Figure 1B and 1C). There were no significant differences between the 10K and 150K fraction when comparing the four markers CD63, TF, Hb and mir-222. The CD63 and TF markers did not differ between controls and Hb STBMs. However, the analysis of the gold signal for mir-222 displayed a down regulation after Hb perfusion (Table 2, and Figure 1B and 1C). Also, mir-222 labelling was found in vesicles expressing both CD63 and TF. Interestingly, in a subsequent preparation with gold-labelling of Hb antibodies, TEM revealed that STBMs carried Hb, and vesicles isolated from Hb perfusions carried more Hb than vesicles from the control perfusions (Table 2, and Figure 1D and 1E).

**Table 2:** Transmission electron microscopy (TEM) data, showing the amount of gold labels per square micrometre.

	10K STBM Control	10K STBM Hb	150K STBM Control	150K STBM Hb
**TF**	46±16	58±15	52±13	63±15
**CD63**	31±12	44±15	28±9	41±18
**mir-222**	98±14	52±17	96±15	63±16
**Hb**	9±3	49±17	10±3	51±17

Values are expressed as mean ± standard deviation.

#### Nanoparticle Tracking Analysis

**Figure 2:**
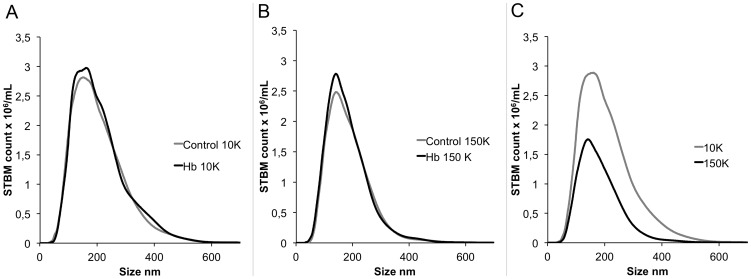
NTA analysis of vesicle size distribution in the 10K and 150K fraction. Nanoparticle tracking analysis (NTA) size distribution profiles for STBMs; comparing effect of Hb perfusion for 10K (A) and 150K (B) STBMs. In (C) the effect of centrifugation speed, 10K vs 150K, is compared. 10K STBMs had a significantly larger median size than 150K STBMs.

To determine the vesicle count and size distribution of vesicles in the 10K and 150K STBMs, we used the NTA methodology. Vesicle count broadly reflected the protein concentration of the 10K STBM preparations (r = 0.71) with stronger correlation in the 150K preparations (r = 0.95). This was probably due to the greater homogeneity observed in the 150K preparations. The size distribution for 10K and 150K STBMs ranged between 50–560 nm and 50–500 nm respectively (Figure 2A and 2B) The size range suggests that both STBM preparations contain both exosomes and microvesicles. There was no difference in this range between the controls and the Hb perfusions. The median size for 10K control was 184 nm and for Hb STBMs 187 nm (Figure 2A). For 150K control and Hb STBMs the median size was 171 nm and 166 nm respectively (Figure 2B). The median size was significantly larger for the 10K STBMs (186 nm) compared to the 150K STBMs (168 nm), shown in Figure 2C. This suggests that the 10K STBMs contain more STBMs in the microvesicle size range and fewer in the exosomes size range.

### Analysis of miRNAs in STBMs

**Figure 3:**
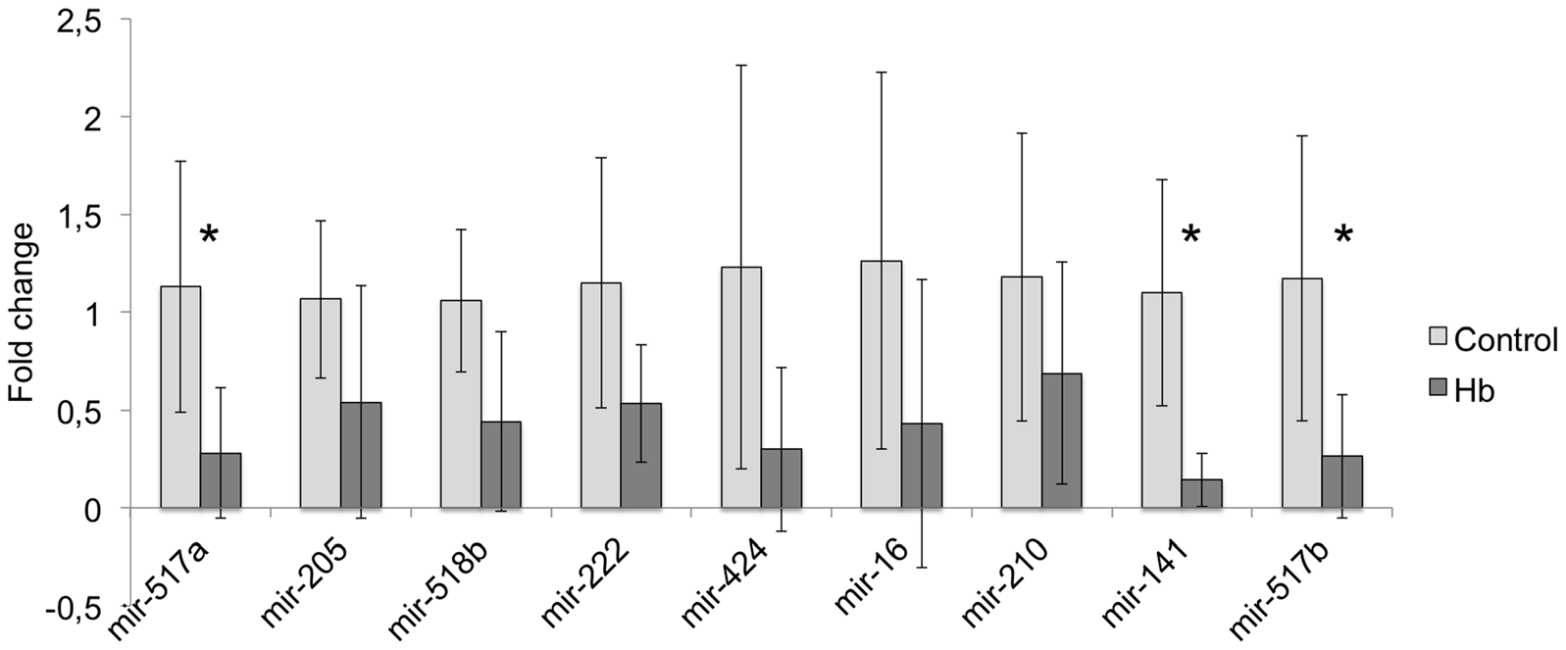
Bar charts showing miRNA fold change in STBMs from control and Hb perfusions. Nine selected miRNAs were analysed using quantitative PCR, as described in the Materials and methods section. The miRNA expression was normalized against Rnu6b and given as fold change. The fold change values were calculated by normalizing against control samples from control perfused placentas. Results are presented as mean ±SD. Differences between the respective control and Hb perfusions were analysed using Mann-Whitney *U*-test. * p<0.05. In the 10K STBMs mir-517a, mir-141 and mir -517b were significantly down regulated in Hb perfusions compared to controls. No significant differences were detected in the 150K STBMs (data not shown).

All miRNAs (mir-517a, mir-517b, mir-518b, mir-205, mir-210, mir-222, mir-141, mir-16 and mir-424) analysed in this study were present in both 10K and 150K STBMs. After Hb perfusion, mir-517a (p = 0.03671), mir-141 (p = 0.01219) and mir-517b (p = 0.03671) were significantly down regulated in 10K STBMs (Figure 3). To confirm that the differences obtained between the groups were dependent on Hb perfusion, mir-141 and mir-517a were also analysed in phase I, before addition of Hb. There was no significant difference (p = 0.27034 and 0.17791 respectively) in phase I. In contrast, the 150K STBMs showed a general trend towards up-regulation of miRNA after Hb perfusion, although none were significant (data not shown).

## Discussion

Extracellular HbF has been suggested as a potential link between the first and second stage of PE [5], [10]. *Ex-vivo* studies have shown that cell-free Hb induces placental damage similar to that seen in PE placentas, and therefore might provide an experimental *ex-vivo* model for PE. Electron microscopy showed that cell-free Hb causes oxidative stress, apoptosis and extensive membrane damage to perfused placentas [6]. In this study, we have further investigated the perfusion medium from these experiments in order to see how cell-free Hb affects the release of STBMs and their miRNA content. The data show that Hb perfusion indeed does alter the content of miRNAs in STBMs. Since perfusion with Hb leads to increased cell blebbing [6] we also hypothesized that the placenta released more STBMs, as described in PE [9], [16], [24]. However the NTA analysis could not confirm this hypothesis. An unexpected and interesting finding was however that the STBMs carried Hb, possibly inside but also on the surface.

The different STBM fractions, showed a decreased median vesicle size in the 150K STBMs compared to the 10K STBMs, confirming earlier results [46]. This suggests more vesicles in the exosome size range (<100 nm) and fewer of microvesicle size range (>100 nm) after higher centrifugation speed. The two surface markers TF and CD63 used in TEM, showed a similar distribution between the two centrifugation fractions. In contrast to the NTA analysis, this finding suggest that there is no major differences between the types of vesicles isolated at different centrifugation speeds. However, even though the surface marker CD63 has been suggested to be a specific exosomes marker [14], it has also been found on the syncytiotrophoblast surface [51], indicating that it is likely to be present on the microvesicles released by syncytiotrophoblasts. The TEM showed that STBMs, larger than 100 nm, were positive for CD63, confirming this. The TF marker was also shown on vesicles of all sizes confirming previous findings [50].

Although there were no obvious differences in the STBM characteristics between control and Hb perfused placentas, we found significant differences in their miRNA content. The nine selected miRNAs were related to the C19MC cluster, hypoxia, PE or Hb synthesis [36], [38]–[45].

Three miRNAs were down regulated in the 10K STBMs after Hb treatment; mir-517a, mir-141 and mir-517b. Mir-141 is one of the most abundant miRNAs in the placenta and found in high levels in maternal plasma during pregnancy [52], [53]. Mir-517a and mir-517b belong to the C19MC cluster [32]. The C19MC miRNAs have previously been shown to be transported by trophoblast exosomes [32], [36]. Recently it has been shown that mir-517b can be transported by trophoblast exosomes to recipient cells, normally not expressing C19MC miRNAs, and inhibit viral infections [54]. By sending out placenta specific miRNAs, the placentas may communicate to the maternal system. By altering the miRNA content in the STBMs, different signals can be sent to the receiving cells.

The TEM analysis showed that mir-222 was down regulated in both 10K and 150K STBMs. This was not confirmed by quantitative PCR, possibly due to small groups. There is an interesting connection between mir-222 and PE, it has been shown to be up-regulated in the PE placenta [38]–[41] but also present in circulating EVs from healthy controls [55]. To our knowledge, no previous studies have described mir-222 in STBMs. The mir-222 regulates fms-like tyrosine kinase-1 (Flt1) [43], which is an anti-angiogenic factor, well described in PE [4]. Furthermore, mir-222 plays a role in the human haemoglobin switch, i.e. when the newborn baby switches from HbF to HbA production, which takes place during the peri/post-natal period [43]. Since previous data have shown an increased production of HbF in PE placentas [5], and elevated levels of s-Flt [4], the role of mir-222 needs further exploration.

The 150K STBMs did not show any significant changes in the miRNA content for the nine miRNAs studied. Data from the NTA analysis suggest that the 150K fractions contain smaller STBMs, and possibly therefore more exosomes. Since exosomes are released by exocytosis and microvesicles by blebbing of the cell surface [12], it may be likely that they load RNA and miRNA in different ways. It has been suggested that exosomes are beneficial to normal pregnancy whereas microvesicles may be harmful [56]. When comparing different trophoblastic cells and cell lines, a previous study has shown great variation of miRNA expression, in particular C19MC miRNAs, which may account for the differences in behaviour between these cells [34]. The differences in miRNA expression between the 10K and 150K fractions might be part of the explanation of why exosomes and microvesicles play different roles in normal pregnancies and in PE. Even though Hb treatment does not alter the shedding of vesicles from the human placenta, the data shows that miRNA content can be significantly altered. This could suggest that Hb has an effect at the level of gene expression. On the other hand, there was a trend towards a generally impoverished miRNA content in 10K STBMs and enriched miRNA content in 150K STBMs. Rather than having an effect on a transcriptional level in the cells, Hb could be changing the profile of STBMs carrying miRNAs where exosomes increase their miRNA content during stress and microvesicles decrease theirs.

The TEM results indicated that cell-free Hb was accumulated inside or on the surface of the STBMs. The small amount of Hb present in the control STBMs, may be due to the natural Hb metabolism occurring in the placenta. During Hb perfusion there is a high Hb concentration outside the vesicles, which may cause binding to the surface. It is important to note that the Hb antibody used for the specific TEM, targets the alpha-chain and therefore measures total Hb, both the Hb added to the perfusion as well as the endogenous foetal production in the placenta. These findings suggest a novel way for Hb to be transferred into the maternal circulation from the placenta, and will be subject for future studies.

In summary, the data in this study suggest that Hb perfusion of the placenta significantly affects the content of some miRNAs in released STBMs. The increased amount of STBMs in PE may be potentially loaded with Hb and differentially expressed miRNAs, which will have negative effects on target cells such as endothelial cells and lymphocytes. It may contribute to the endothelial dysfunction and inflammation seen in PE [4]. STBMs may be important for communicating the status of the placenta systemically. Accumulation of Hb in STBMs may prevent Hb from being degraded. Upon fusion with other cell types, a direct intracellular deposit of Hb may cause toxic damage. Accumulated Hb may also oxidize the STBMs content, modifying RNA and proteins, which could have further impact on the vesicle-to-cell signalling.

## References

[1] Sibai B, Dekker G, Kupferminc M (2005) Pre-eclampsia. Lancet 365: 785–799. 15733721 10.1016/S0140-6736(05)17987-2

[2] Milne F, Redman C, Walker J, Baker P, Bradley J, et al. (2005) The pre-eclampsia community guideline (PRECOG): how to screen for and detect onset of pre-eclampsia in the community. BMJ. England. pp. 576–580. 10.1136/bmj.330.7491.576 PMC554032 15760998

[3] Duley L, Meher S, Abalos E (2006) Management of pre-eclampsia. BMJ 332: 463–468. 16497761 10.1136/bmj.332.7539.463 PMC1382544

[4] Redman CW, Sargent IL (2005) Latest advances in understanding preeclampsia. Science 308: 1592–1594. 15947178 10.1126/science.1111726

[5] Centlow M, Carninci P, Nemeth K, Mezey E, Brownstein M, et al (2008) Placental expression profiling in preeclampsia: local overproduction of hemoglobin may drive pathological changes. Fertil Steril 90: 1834–1843. 18166190 10.1016/j.fertnstert.2007.09.030 PMC2628488

[6] May K, Rosenlof L, Olsson MG, Centlow M, Morgelin M, et al (2011) Perfusion of human placenta with hemoglobin introduces preeclampsia-like injuries that are prevented by alpha1-microglobulin. Placenta 32: 323–332. 21356557 10.1016/j.placenta.2011.01.017

[7] Faivre B, Menu P, Labrude P, Vigneron C (1998) Hemoglobin autooxidation/oxidation mechanisms and methemoglobin prevention or reduction processes in the bloodstream. Literature review and outline of autooxidation reaction. Artif Cells Blood Substit Immobil Biotechnol 26: 17–26. 9507753 10.3109/10731199809118943

[8] Olsson MG, Centlow M, Rutardottir S, Stenfors I, Larsson J, et al (2010) Increased levels of cell-free hemoglobin, oxidation markers, and the antioxidative heme scavenger alpha(1)-microglobulin in preeclampsia. Free Radic Biol Med 48: 284–291. 19879940 10.1016/j.freeradbiomed.2009.10.052

[9] Reddy A, Zhong XY, Rusterholz C, Hahn S, Holzgreve W, et al (2008) The effect of labour and placental separation on the shedding of syncytiotrophoblast microparticles, cell-free DNA and mRNA in normal pregnancy and pre-eclampsia. Placenta 29: 942–949. 18834630 10.1016/j.placenta.2008.08.018

[10] Anderson UD, Olsson MG, Rutardottir S, Centlow M, Kristensen KH, et al. (2011) Fetal hemoglobin and alpha1-microglobulin as first- and early second-trimester predictive biomarkers for preeclampsia. Am J Obstet Gynecol 204: 520 e521–525. 10.1016/j.ajog.2011.01.058 21439542

[11] Roos MA, Gennero L, Denysenko T, Reguzzi S, Cavallo G, et al (2010) Microparticles in physiological and in pathological conditions. Cell Biochem Funct 28: 539–548. 20941744 10.1002/cbf.1695

[12] Chaput N, Thery C (2011) Exosomes: immune properties and potential clinical implementations. Semin Immunopathol 33: 419–440. 21174094 10.1007/s00281-010-0233-9

[13] Cocucci E, Racchetti G, Meldolesi J (2009) Shedding microvesicles: artefacts no more. Trends Cell Biol 19: 43–51. 19144520 10.1016/j.tcb.2008.11.003

[14] Mincheva-Nilsson L, Baranov V (2010) The role of placental exosomes in reproduction. Am J Reprod Immunol 63: 520–533. 20331583 10.1111/j.1600-0897.2010.00822.x

[15] Orozco AF, Lewis DE (2010) Flow cytometric analysis of circulating microparticles in plasma. Cytometry A 77: 502–514. 20235276 10.1002/cyto.a.20886 PMC2919894

[16] Aharon A, Brenner B (2011) Microparticles and pregnancy complications. Thromb Res 127 Suppl 3S67–71. 21262446 10.1016/S0049-3848(11)70019-6

[17] Orozco AF, Jorgez CJ, Ramos-Perez WD, Popek EJ, Yu X, et al (2009) Placental release of distinct DNA-associated micro-particles into maternal circulation: reflective of gestation time and preeclampsia. Placenta 30: 891–897. 19692120 10.1016/j.placenta.2009.06.012 PMC2758063

[18] Valadi H, Ekstrom K, Bossios A, Sjostrand M, Lee JJ, et al (2007) Exosome-mediated transfer of mRNAs and microRNAs is a novel mechanism of genetic exchange between cells. Nat Cell Biol 9: 654–659. 17486113 10.1038/ncb1596

[19] Yuan A, Farber EL, Rapoport AL, Tejada D, Deniskin R, et al (2009) Transfer of microRNAs by embryonic stem cell microvesicles. PLoS One 4: e4722. 19266099 10.1371/journal.pone.0004722 PMC2648987

[20] Alijotas-Reig J, Palacio-Garcia C, Llurba E, Vilardell-Tarres M (2013) Cell-derived microparticles and vascular pregnancy complications: a systematic and comprehensive review. Fertil Steril 99: 441–449. 23122952 10.1016/j.fertnstert.2012.10.009

[21] Dechend R, Staff AC (2012) Placenta messages to the mother: not just debris. Hypertension 59: 191–193. 22215710 10.1161/HYPERTENSIONAHA.111.184861

[22] Quesenberry PJ, Aliotta JM (2010) Cellular phenotype switching and microvesicles. Adv Drug Deliv Rev 62: 1141–1148. 20558219 10.1016/j.addr.2010.06.001 PMC2955803

[23] Messerli M, May K, Hansson SR, Schneider H, Holzgreve W, et al (2010) Feto-maternal interactions in pregnancies: placental microparticles activate peripheral blood monocytes. Placenta 31: 106–112. 20005571 10.1016/j.placenta.2009.11.011

[24] Knight M, Redman CW, Linton EA, Sargent IL (1998) Shedding of syncytiotrophoblast microvilli into the maternal circulation in pre-eclamptic pregnancies. Br J Obstet Gynaecol 105: 632–640. 9647154 10.1111/j.1471-0528.1998.tb10178.x

[25] Sharp AN, Heazell AE, Crocker IP, Mor G (2010) Placental apoptosis in health and disease. Am J Reprod Immunol 64: 159–169. 20367628 10.1111/j.1600-0897.2010.00837.x PMC3025811

[26] Germain SJ, Sacks GP, Sooranna SR, Sargent IL, Redman CW (2007) Systemic inflammatory priming in normal pregnancy and preeclampsia: the role of circulating syncytiotrophoblast microparticles. J Immunol 178: 5949–5956. 17442979 10.4049/jimmunol.178.9.5949

[27] Longtine MS, Chen B, Odibo AO, Zhong Y, Nelson DM (2012) Villous trophoblast apoptosis is elevated and restricted to cytotrophoblasts in pregnancies complicated by preeclampsia, IUGR, or preeclampsia with IUGR. Placenta 33: 352–359. 22341340 10.1016/j.placenta.2012.01.017 PMC3467099

[28] von Dadelszen P, Hurst G, Redman CW (1999) Supernatants from co-cultured endothelial cells and syncytiotrophoblast microvillous membranes activate peripheral blood leukocytes in vitro. Hum Reprod 14: 919–924. 10221219 10.1093/humrep/14.4.919

[29] Aly AS, Khandelwal M, Zhao J, Mehmet AH, Sammel MD, et al (2004) Neutrophils are stimulated by syncytiotrophoblast microvillous membranes to generate superoxide radicals in women with preeclampsia. Am J Obstet Gynecol 190: 252–258. 14749668 10.1016/j.ajog.2003.07.003

[30] Li M, Marin-Muller C, Bharadwaj U, Chow KH, Yao Q, et al (2009) MicroRNAs: control and loss of control in human physiology and disease. World J Surg 33: 667–684. 19030926 10.1007/s00268-008-9836-x PMC2933043

[31] Maccani MA, Marsit CJ (2009) Epigenetics in the placenta. Am J Reprod Immunol 62: 78–89. 19614624 10.1111/j.1600-0897.2009.00716.x PMC2813777

[32] Donker RB, Mouillet JF, Chu T, Hubel CA, Stolz DB, et al (2012) The expression profile of C19MC microRNAs in primary human trophoblast cells and exosomes. Mol Hum Reprod 18: 417–424. 22383544 10.1093/molehr/gas013 PMC3389496

[33] Mouillet JF, Chu T, Sadovsky Y (2011) Expression patterns of placental microRNAs. Birth Defects Res A Clin Mol Teratol 91: 737–743. 21425434 10.1002/bdra.20782 PMC5030720

[34] Morales-Prieto DM, Chaiwangyen W, Ospina-Prieto S, Schneider U, Herrmann J, et al (2012) MicroRNA expression profiles of trophoblastic cells. Placenta 33: 725–734. 22721760 10.1016/j.placenta.2012.05.009

[35] Gu Y, Sun J, Groome LJ, Wang Y (2013) Differential miRNA expression profiles between the first and third trimester human placentas. Am J Physiol Endocrinol Metab 304: E836–843. 23443922 10.1152/ajpendo.00660.2012 PMC3625781

[36] Luo SS, Ishibashi O, Ishikawa G, Ishikawa T, Katayama A, et al (2009) Human villous trophoblasts express and secrete placenta-specific microRNAs into maternal circulation via exosomes. Biol Reprod 81: 717–729. 19494253 10.1095/biolreprod.108.075481

[37] Miura K, Miura S, Yamasaki K, Higashijima A, Kinoshita A, et al (2010) Identification of pregnancy-associated microRNAs in maternal plasma. Clin Chem 56: 1767–1771. 20729298 10.1373/clinchem.2010.147660

[38] Enquobahrie DA, Abetew DF, Sorensen TK, Willoughby D, Chidambaram K, et al. (2011) Placental microRNA expression in pregnancies complicated by preeclampsia. Am J Obstet Gynecol 204: 178 e112–121. 10.1016/j.ajog.2010.09.004 PMC3040986 21093846

[39] Hu Y, Li P, Hao S, Liu L, Zhao J, et al (2009) Differential expression of microRNAs in the placentae of Chinese patients with severe pre-eclampsia. Clin Chem Lab Med 47: 923–929. 19642860 10.1515/CCLM.2009.228

[40] Pineles BL, Romero R, Montenegro D, Tarca AL, Han YM, et al. (2007) Distinct subsets of microRNAs are expressed differentially in the human placentas of patients with preeclampsia. Am J Obstet Gynecol 196: 261 e261–266. 10.1016/j.ajog.2007.01.008 17346547

[41] Zhu XM, Han T, Sargent IL, Yin GW, Yao YQ (2009) Differential expression profile of microRNAs in human placentas from preeclamptic pregnancies vs normal pregnancies. Am J Obstet Gynecol 200: : 661 e661–667. 10.1016/j.ajog.2008.12.045 19285651

[42] Bianchi N, Zuccato C, Lampronti I, Borgatti M, Gambari R (2009) Expression of miR-210 during erythroid differentiation and induction of gamma-globin gene expression. BMB Rep 42: 493–499. 19712585 10.5483/bmbrep.2009.42.8.493

[43] Gabbianelli M, Testa U, Morsilli O, Pelosi E, Saulle E, et al (2010) Mechanism of human Hb switching: a possible role of the kit receptor/miR 221-222 complex. Haematologica 95: 1253–1260. 20305142 10.3324/haematol.2009.018259 PMC2913072

[44] Sankaran VG (2011) Targeted therapeutic strategies for fetal hemoglobin induction. Hematology Am Soc Hematol Educ Program 2011: 459–465. 22160074 10.1182/asheducation-2011.1.459

[45] Mouillet JF, Chu T, Nelson DM, Mishima T, Sadovsky Y (2010) MiR-205 silences MED1 in hypoxic primary human trophoblasts. FASEB J 24: 2030–2039. 20065103 10.1096/fj.09-149724 PMC2874470

[46] Guller S, Tang Z, Ma YY, Di Santo S, Sager R, et al (2011) Protein composition of microparticles shed from human placenta during placental perfusion: Potential role in angiogenesis and fibrinolysis in preeclampsia. Placenta 32: 63–69. 21074265 10.1016/j.placenta.2010.10.011 PMC3762591

[47] Oehmcke S, Morgelin M, Malmstrom J, Linder A, Chew M, et al (2012) Stimulation of blood mononuclear cells with bacterial virulence factors leads to the release of pro-coagulant and pro-inflammatory microparticles. Cell Microbiol 14: 107–119. 21951918 10.1111/j.1462-5822.2011.01705.x

[48] Bober M, Enochsson C, Collin M, Morgelin M (2010) Collagen VI is a subepithelial adhesive target for human respiratory tract pathogens. J Innate Immun 2: 160–166. 20375633 10.1159/000232587

[49] Dragovic RA, Gardiner C, Brooks AS, Tannetta DS, Ferguson DJ, et al (2011) Sizing and phenotyping of cellular vesicles using Nanoparticle Tracking Analysis. Nanomedicine 7: 780–788. 21601655 10.1016/j.nano.2011.04.003 PMC3280380

[50] Gardiner C, Tannetta DS, Simms CA, Harrison P, Redman CW, et al (2011) Syncytiotrophoblast microvesicles released from pre-eclampsia placentae exhibit increased tissue factor activity. PLoS One 6: e26313. 22022598 10.1371/journal.pone.0026313 PMC3194796

[51] Hedlund M, Stenqvist AC, Nagaeva O, Kjellberg L, Wulff M, et al (2009) Human placenta expresses and secretes NKG2D ligands via exosomes that down-modulate the cognate receptor expression: evidence for immunosuppressive function. J Immunol 183: 340–351. 19542445 10.4049/jimmunol.0803477

[52] Chim SS, Shing TK, Hung EC, Leung TY, Lau TK, et al. (2008) Detection and characterization of placental microRNAs in maternal plasma. Clin Chem. United States. pp. 482–490. 10.1373/clinchem.2007.097972 18218722

[53] Mouillet JF, Chu T, Hubel CA, Nelson DM, Parks WT, et al. (2010) The levels of hypoxia-regulated microRNAs in plasma of pregnant women with fetal growth restriction. Placenta. England: 2010 Elsevier Ltd. pp. 781–784. 10.1016/j.placenta.2010.07.001 PMC3204658 20667590

[54] Delorme-Axford E, Donker RB, Mouillet JF, Chu T, Bayer A, et al (2013) Human placental trophoblasts confer viral resistance to recipient cells. Proc Natl Acad Sci U S A 110: 12048–12053. 23818581 10.1073/pnas.1304718110 PMC3718097

[55] Hunter MP, Ismail N, Zhang X, Aguda BD, Lee EJ, et al (2008) Detection of microRNA expression in human peripheral blood microvesicles. PLoS One 3: e3694. 19002258 10.1371/journal.pone.0003694 PMC2577891

[56] Redman CW, Tannetta DS, Dragovic RA, Gardiner C, Southcombe JH, et al (2012) Review: Does size matter? Placental debris and the pathophysiology of pre-eclampsia. Placenta 33 SupplS48–54. 22217911 10.1016/j.placenta.2011.12.006

